# Can Citizen Science Be a Key Factor in the Fight Against Mislabeling? Discovering What Squid Is on the Plate

**DOI:** 10.3390/foods15101690

**Published:** 2026-05-12

**Authors:** Marta Muñoz-Colmenero, Marta Vargas-Ramírez, Pedro Perdiguero, Isabel Ballesteros, Beatriz Beroiz, Félix Gil, Rosario Linacero, Jose Luis Horreo

**Affiliations:** Department of Genetics, Physiology and Microbiology, Faculty of Biological Sciences, Complutense University of Madrid, 28040 Madrid, Spain

**Keywords:** citizen science, food control, mislabeling, squids, *Loligo* spp.

## Abstract

Governments invest much effort trying to control food markets as well as to protect consumer rights, but resource limitations and the complexity of the scenario make it a challenging task. To overcome this, the use of Citizen Science could potentially be a powerful tool to fight against food fraud. We have researched this through the assessment of mislabeling for squids of *Loligo* genus, one of the most appreciated and consumed foods in Spain and Europe, with a big information gap regarding their mislabeling status. We involved volunteers to collect squid samples at Spanish restaurants, genetically identifying them later. Thanks to participants, sampling was highly successful, with 93.55% of the samples recovered and very similar mislabeling values being found whether samples were collected by experts or volunteers. Citizen Science provided high territorial coverage (82% of Spanish autonomous communities) to gain a comprehensive view of *Loligo* mislabeling in Spanish restaurants, its magnitude (91.59%) and consumer preferences, demonstrating a huge potential of the use of Citizen Science in traceability studies. Interestingly, mislabeling was equally present in both sandwiches and plates, was higher in inland localities than on the coast, and was higher in cheaper products. Participants reported a greater understanding of the concept of mislabeling after their participation and expressed high interest in their involvement in scientific projects in the future. They also emphasized the importance of fostering a close relationship between science and society.

## 1. Introduction

Food fraud is a worldwide problem that can cause economic, sustainability, and potential food safety problems for consumers, governments regulatory authorities, and the food industry [1]. Far from decreasing, the number of food fraud cases are constantly increasing, which can be partially attributed to the current international trade, where products from any part of the world are easily accessible. There are new markets with different food prices, control systems, and norms all around the world that result in problems to control and verify compliance with food regulations in an international context [2].

There are two main concerns related to food fraud focused on corroborating that no mislabeling or substitutions exist, and that the origin and the ingredients of the products are correctly detailed: adulteration and authenticity [3]. Several cases of mislabeling have been reported in the literature over time and in a large variety of products [4,5,6,7,8,9,10]. Furthermore, given the complexity of controlling the food market in a globalized and interconnected world, it is expected that this will continue, so establishing effective control measures becomes essential. Countries and their governments continue to invest money, efforts and resources in legislating and trying to protect the natural resources that are affected by this problem, as well as protecting consumer rights. However, in many cases, the limited economic, logistical, technical, and human resources available to authorities hinder effective implementation, both in more developed and less developed countries [2,11]. Spain is no exception to this, where controls are scarce and often focused on a few specific products each year due to the reduced funding available, being insufficient for adequate control of the wide variety of products subject to fraud. Added to this resource deficit, food services present more complexity for their control, as it is not mandatory for European members to disclose key information like scientific names of the species on their menus unless specifically requested by the competent authorities, which generates even more difficulties in food control and transparency of information for the consumer [12].

It is perhaps at this point where Citizen Science could potentially reinforce these control systems. Citizen Science (CS) is defined as a collaborative scientific methodology that bridges the gap between the academic community and society by engaging volunteer citizens in the systematic generation of new knowledge. Within the framework of food authenticity and traceability, this approach functions as a strategic mechanism for large-scale data acquisition, mobilizing the public to collect vast quantities of samples or information (including mislabeling; [13]), as well as disseminating results or important scientific information among the public and engaging citizens in scientific research [14], but rarely both things at the same time. The use of CS in scientific projects of high interest for society and its well-being (as is the control of the food system) seems to offer wide benefits. Although the most used analytical techniques to detect mislabeling have become more affordable, the price of food products has increased [15], requiring the sampling of a large part of the investment available for fraud control campaigns to reach a representative number of samples, and thus CS sampling could also be helpful in this aspect. Some studies on food fraud have already taken advantage of this, yielding interesting and useful results for science and society by using CS [7,12,13,16,17], describing the citizen participation as an integral part of their successful campaigns and essential to advance traceability policies with decision makers. In relation to this, a legitimate concern regarding the use of Citizen Science is the reliability of the data and the ability of CS data to answer scientific research questions. However, many studies have confirmed a high level of Citizen Science data quality providing information on the scientific outcomes (e.g., review [18]). Along this line, we have used the CS (volunteers) as “samplers” of squids (calamari) sold and consumed cooked at restaurants in Spain.

Among food fraud cases, fish species substitution has been widely reported, but there is a lack of information regarding commercial crustaceans and cephalopod species [19,20] (see below). To diminish somehow this gap, we chose the squids, because they are highly consumed and appreciated in Europe [21] and specifically in Spain [22]. Some previous studies about seafood mislabeling have included squid samples; for example, Guardone et al. [23], who conducted a survey on labeling non-compliance in fishery products imported from extra-European countries at one Border Inspection Post of Italy, finding 43.8% mislabeling in cephalopods. Pardo et al. [22] detected a 60% mislabeling rate for cephalopods in European caterings, primarily for *Loligo* and *Octopus* genera, and Pardo and Jiménez [12] found 57% mislabeling for squids in food services, with a significant mislabeling identified in the cases labeled as “calamar/chipirón”, but the number of collected samples was low and not randomly collected in both cases. Giagkazoglou et al. [21] found in Greece that most mislabeled samples of their study from restaurants (37 out of 59) were squids, with many cases strongly suspected to be intentional fraud. Lately, Sotelo et al. [24] assessed the mislabeling rate in processed squid products sold in Spanish markets, finding a low mislabeling rate (11.1%) but a wide variety of cephalopod species being used as substitutes, and there was a wide range of brand names. In line with the recommendations made by Luque and Donlan [19], suggesting that the estimation of the mislabeling rate is more efficient and realistic when the studies are focused only on specific products and in only one genus or species, we decided to drive the volunteers forward to take from restaurants only those samples that declared “calamar” or “chipiron” on the menu. According to the official list of commercial names of fishery products marketed in Spain, updated in 2023 by the Spanish Ministry (https://www.mapa.gob.es/es/pesca/temas/mercados-economia-pesquera/listado-denominaciones-comerciales-anexo-unico-actualizado-a-marzo-de-2023_tcm30-645033.pdf; last accessed on 22 October 2024), these products must only contain *Loligo* species, except when some other clarification about the species can be read on the menu.

Since the current mislabeling for *Loligo* genus in Spanish restaurants remains unknown despite it being one of the countries with the highest consumption, we have set out two main objectives in this work: (i) to assess the rate of mislabeling of the genus *Loligo* in Spanish restaurants, where they are highly appreciated and consumed, its prevalence, and the diversity of species found, and (ii) to evaluate the potential of a Citizen Science force as a tool for controlling mislabeling and combating this type of food fraud. In recent years, the focus of food fraud prevention has shifted from assessing exposure to danger to evaluating the likelihood of the risk [3] and we believe that Citizen Science is perfect to assess this risk. Understanding the scope of the problem is essential for implementing preventive measures and finding solutions, and this study will be the first step toward regulating the market for *Loligo* squid in restaurants.

## 2. Materials and Methods

### 2.1. Sampling

All samples were collected in different bars and restaurants of Spain, from June 2023 to January 2024. Only the samples named as “calamar”, “calamares” or “chipirón” on the menu were sampled, following current Spanish legislation (BOE-A-2019-9026). To evaluate the utility of Citizen Science to collect samples in studies of food traceability, the samples were collected mostly by volunteers and some of them were collected by the researchers involved in the work to compare both results.

**Figure 1:**
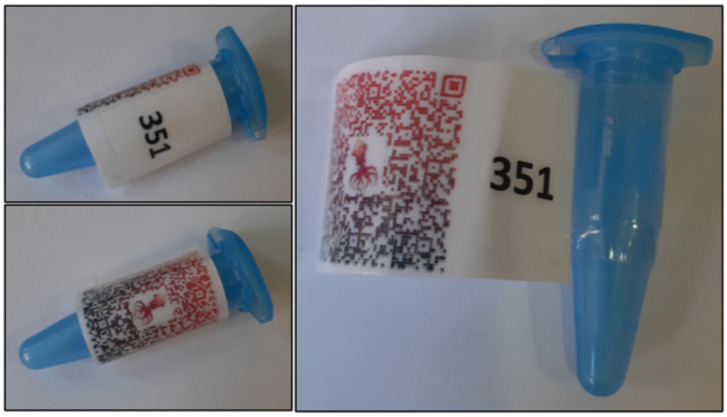
Tubes for sampling with the number ID and the QR code linked to the Google form. Note that the QR code included a squid in the center.

Information about the project was shared among volunteers by word of mouth, and interested volunteers participated voluntarily and without receiving any incentive or compensation for their participation. Each volunteer interested in participation received a tube with a number and a barcode linked with an anonymous Google form (Figure 1). That form had an introduction where the volunteers were informed about what “mislabeling” actually means and the objective of the collaborative sampling. Additionally, information about how to collect and store (frozen) the samples until delivery was also available there. The form was divided into two parts: the first one, mandatory, was where the volunteers included the information about the sample. Specifically, they were asked about: number of the tube, date, name of bar/restaurant, location, name of the food on the menu, type of food (sandwich or plate of calamari), cooking form (battered/non-battered or grilled/in its ink), and price. As additional information and optional, we asked about their name/nickname and email contact, this last in order to inform them about the results of the project in the near future.

The second part of the Google form, which also was optional, was a questionnaire that asked about four concepts: (i) statistical data society ranks (questions p1 and p2), (ii) feelings about participating in this project (p3–p7), (iii) knowledge about mislabeling (p8–p10), (iv) their opinion about the current relationship between the scientific community and society, and a final question about measures that could improve the relationship between them (p11–p13). This questionnaire is disclosed in Supporting Materials S1.

The samples were collected at a central drop-off point located in the Department of Genetics, Physiology, and Microbiology at the Complutense University of Madrid. For locations outside the Autonomous Community of Madrid, researchers went to collect the samples at an agreed-upon location whenever possible.

### 2.2. Evaluation of Sampling Success

In order to be able to evaluate the quality of data and samples recovered, some parameters were considered: (1) percentage of samples recovered with complete information, (2) percentage of samples for which good quality DNA sequences were obtained, and (3) evaluation of the success of PCR and mislabeling. These data were evaluated globally and as two separated groups: (i) samples collected by experts from our department and (ii) those collected by volunteers, in order to compare whether the results are similar, following the methodology applied by Warner et al. [16].

On the other hand, to determine whether CS could be a viable option for future studies of mislabeling or fraud and/or government control campaigns, the success of the sampling was evaluated attending to two main aspects: the number of samples recovered with complete information (tube with sample piece plus sample information in the Google form) and the geographical coverage (how many different autonomous communities, provinces, and localities we got samples from).

### 2.3. DNA Extraction, PCR Amplification, and Sequence Analyses

Nucleic acid isolation was performed with a standardized protocol using ammonium acetate [25] from a piece of 2–4 mm^3^ of tissue. The amplification of the cytochrome oxidase subunit I gene (COI) fragment was carried out in a SensoQuest LabCycler PCR machine (Progen Scientific Ltd., London, UK), using the pair of primers described by Folmer et al. [26]: LCO-1490: 5′-GGTCAACAAATCATAAAGATATTGG-3 and HCO-2198: 5′-TAAACTTCAGGGTGACCAAAAAATCA-3, adding 1 µL of each one (5 µmol/L). The PCR reaction was performed using the Amplitools Master Mix (Biotools S.A., Madrid, Spain) and with 2 µL of the DNA previously isolated. PCR conditions were an initial denaturation at 95 °C for 5 min followed by 35 cycles of 30 s at 94 °C, 30 s at 48 °C for annealing and 30 s at 72 °C for elongation, and a final extension step of 72 °C for 5 min. PCR products were visualized on 1.8% agarose gels stained with Sybr Safe DNA Gel Stain (Invitrogen, Carlsbad, CA, USA: ref. S33102). The products that got positive results in the gel were sequenced and analyzed in the ABI PRISM 3130XL sequencer at the Genomic Unit of the UCM.

Sequences were edited with BioEdit v 7.7.1 Sequence Alignment Editor Software [27]. To identify the species, they were compared using the nucleotide BLAST on-line tool (nBLAST; https://blast.ncbi.nlm.nih.gov/Blast.cgi; last accessed on 22 October 2024) within the GenBank database from NCBI (National Center for Biotechnology Information, Bethesda, MD, USA; http://www.ncbi.nlm.nih.gov/; last accessed on 22 October 2024). The threshold to consider a successful identification was set up in 99%, following Patil et al. [28]. Additionally, the second species that appeared in the BLAST following % similarity was also noted, checking that the similarity suffered a significant decrease or not.

### 2.4. Labeling Regulations, Mislabeling, and Statistical Analyses

The European Regulation (EU) 1379/2013 establishes what information must be declared when a seafood product is sold. As part of this regulation, each EU Member State must publish an official list with the official trade names, including scientific, local, or regional names in the different official languages of the country. In the case of Spain, the Ministry of Agriculture, Fishing, and Food published on 24 May 2019 the list with the official trade names for the seafood species (BOE-A-2019-9026). Additionally, this list is updated every year, the latest version having been published on 3 February 2023 (https://www.mapa.gob.es/es/pesca/temas/mercados-economia-pesquera/listado-denominaciones-comerciales-anexo-unico-actualizado-a-marzo-de-2023_tcm30-645033.pdf; last accessed on 22 October 2024). According to that list, when the name in the menu is “calamar” or “chipirón”, the expected species are those belonging to the genus *Loligo* spp., so all samples in which the species were determined after the genetic analyses as being from other genera were classified as mislabeling.

The statistical analyses were performed with R Studio v. 3.6.3. A Chi-square (χ^2^) test of independence was applied to determine whether the mislabeling rates differed significantly between categories, such as sandwiches versus plates of calamari, or coastal versus inland localities. For analyses related to the price of samples, the descriptive parameters (mean, median, range and standard deviation) were calculated. To check if the differences in price were significant between categories, a *t*-test was performed, considering the homogeneity of variances or not.

### 2.5. Analyses of the Questionnaire

Each part of the questionnaire was first analyzed separately. The data society ranks, age, and educational level were recovered from the Google form. For the second part, feelings about participating in this project, the five questions included were designed as a set of Likert-based items, with values from 1 to 5, and the counts for each value were noted. In the case of the third part, asking about mislabeling knowledge, the three items were different. The question p8 was also based on a Likert scale with values from 1 to 5 and the answers were counted. For p9, related to the number of cases of mislabeling that the person knew about, five possible answers were proposed (Supporting Materials S1), with a middle answer “to know at least one case of mislabeling” being interpreted as having some knowledge about mislabeling, and the last two options (to know two to five cases of mislabeling or more than five) were interpreted as quite a bit of knowledge about mislabeling. The last question, p10, also showed five specific options to answer related to their knowledge about mislabeling after participating in this project. In this case, any of the last three answer options were considered as a positive sequential increase in their knowledge about mislabeling, while the two first ones were interpreted as no increment in knowledge had been obtained from participation. Finally, for the fourth part, asking about their assessment of the current relationship between science and society and possible actions to take, p11 and p12 were evaluated with four possible answers for each one. Both questions were related to the objective of evaluating if the current contact and transfer of information between science and society are efficient, from the point of view of society, as well as how it should be according to the citizens’ opinions. The last question (q13.) consisted of a list of six different actions that could improve communication and the relationship between the scientific community and society. Additionally, an “other” category was also included in this question, where the person could write a new proposal for action. In this question it was valid to mark as many options as were considered important. When analyzed, the results were tallied. All data were noted and shown in different graphs grouped by part.

## 3. Results

### 3.1. Citizen Science: Sampling Success

A total of 124 tubes were recovered after the sampling period. From those tubes, 116 (93.55%) had information associated with the sample reported in the Google form, so those samples were considered “complete” and included in our genetic analyses, involving 86 people (79 citizen and 7 experts). On the other hand, there also were six people that filled in the Google form but did not return the tube with the sample (4.92%). From complete samples, 107 samples (92.24%; 88% in the case of samples collected by experts (N = 22) and 93.41% for volunteers (N = 85); χ^2^ = 0.80, d.f. = 1, *p*-value > 0.05) resulted in a visible PCR product and clean and good quality sequence of DNA and these were considered to evaluate the sampling success in coverage.

We obtained samples from 14 out of the 17 autonomous communities (82%) present in Spain (excluding Ceuta y Melilla autonomous cities) and from 25 out of 50 provinces (50%) (Supporting Materials S2: Figure S1). These samples were distributed in 48 different localities (Supporting Materials S2: Table S1), which varied greatly in size, as measured by population. Fifteen of the 48 localities were small towns and villages with populations of fewer than 10,000.

Regarding the sample type, 103 samples showed “calamar” on the menu and only four the “chipirón” denomination. Most of the samples were from a plate of calamari (83), with three types of cooking, battered and fried (68), grilled (7), or ink (1). The rest of the samples were sandwiches of battered calamari (24). Therefore, all the common types of calamari presentation were sampled and with expected proportions, since the preferred and most common tapa of calamari in Spain is that battered and fried.

### 3.2. Mislabeling Results

The percentages of identity with the sequences in the GenBank database were between 99.35–100% for all cases and the coverage never was lower than 95%; the e-value was 0.0 for all of them (Supporting Materials S2: Table S2). Additionally, the second species that matched in the NCBI BLAST was checked (Supporting Materials S2: Table S2) and the percentages of identity for this second species match were from 84.14–97.06%. Only in eight cases, in which the first species match was *Illex argentinus*, did the second species that matched obtain a percentage of identity higher than 99%, but in these cases the coverage fell to 87–90%; in these cases, the third species that matched always was *Nototodarus gouldi* or *Todarodes pacificus*, but never *Loligo* genus.

**Figure 2:**
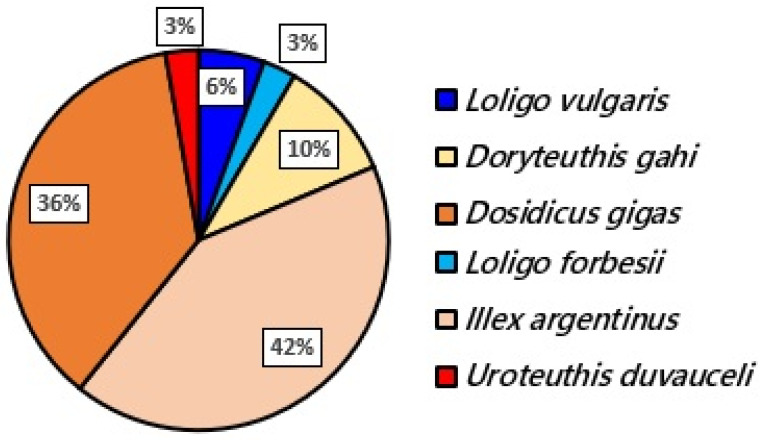
Species found after genetic analyses of the samples. All species but *Loligo* species implied mislabeling.

Only nine samples of the 107 samples were *Loligo* spp.: six were *Loligo vulgaris* (European squid or common squid), and three *Loligo forbesii* (veined squid and long-finned squid). This makes a total percentage of squid mislabeling in Spain of 91.59%. This mislabeling rate was high and similar for both groups of collectors, experts (95.45%) and volunteers (90.59%) (χ^2^ = 0.54, d.f. = 1, *p*-value > 0.05). The non-*Loligo* species belonged to four genera and four different species, *Illex argentinus* (Argentine shortfin squid), *Dosidicus gigas* (Humboldt squid or Jumbo flying squid), *Doryteuthis gahi* (Patagonian longfin squid), and *Uroteithis duvauceli* (Indian Ocean squid), following an increasing order of presence (Figure 2). All these species have a Spanish official commercial name disclosed in the list associated to the proper regulation and updated in 2023, except the last one, which does not appear in the document as a possible commercial species in Spain.

**Figure 3:**
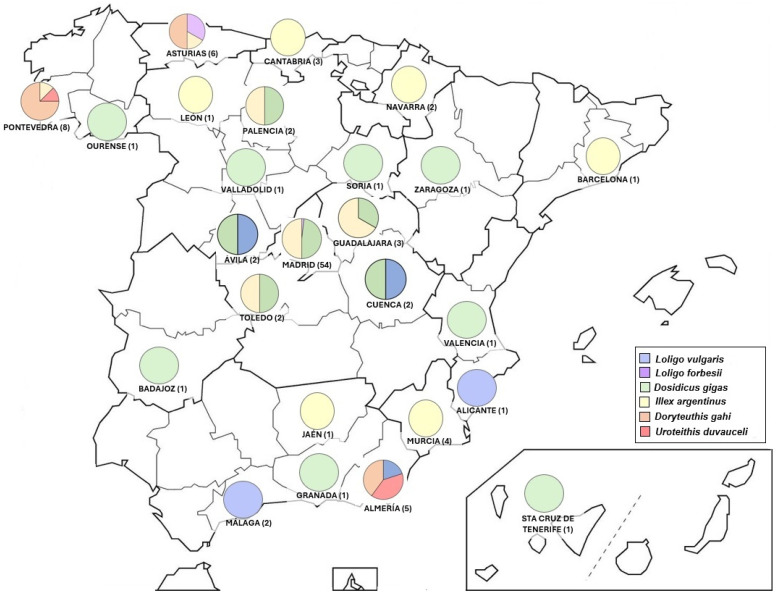
Distribution of the species found in the different provinces sampled. The number of the samples in each province is indicated in parenthesis.

Regarding species distribution across Spain, the *Loligo* species were found mainly in coastal localities. In fact, the mislabeling on the coast was significantly lower (80%) than in the inland localities (96.1%) (χ^2^ = 17.82, d.f. = 1, *p*-value < 0.05). In the same way, excluding Madrid, because it is the capital of the country and the province with more samples collected, the mislabeling in the southern localities was significantly lower (75%) than in the north (90.9%) (χ^2^ = 4.87, d.f. = 1, *p*-value < 0.05). *L. forbesii* was found only in the north (Asturias) and one only sample in Madrid (Figure 3). *L. vulgaris*, however, appeared mainly in southern and southeastern provinces and two loose samples in the center of the peninsula (Ávila and Cuenca) (Figure 3). The two species most used as substitutes were *D. gigas*, which was less present in coastal areas but homogeneously spread in inland regions, and *I. argentinus*, which was more used in the north and even more in coastal localities (Figure 3). *D. gahi* was found only in three provinces, two in the north (Pontevedra and Asturias) and one in the south (Almería), all of them in coastal localities. Lastly, *U. duvauceli* was found in two of those coastal provinces, Pontevedra and Almería.

With respect to the types of food, no significant differences were found between sandwich (95.8%) and plate of calamari (90.4%) (χ^2^ = 0.725, d.f. = 1, *p*-value > 0.05). All the species were found in both types except *L. forbesii* and *D. gahi* which were not found in sandwiches. With respect to the plate category, mislabeling was 92.6% for battered and fried samples and 71.4% for grilled samples. The only sample that was cooked in its ink resulted in identification as *L. vulgaris*. Due to the disparity in the number of samples for each cooking category, no statistical test was made.

Regarding the price of samples (Table 1), this was significantly higher for the few non-mislabeled samples than for mislabeled samples. As was expected, the price for plates was significantly higher than for sandwiches. In the case of plate samples, the price for non-mislabeled samples was significantly higher too. For sandwich samples, this pattern was repeated but no statistical test was carried out due to only one sample not being mislabeled.

### 3.3. Citizen Science: Participation and Opinion About Science and Transference

**Figure 4:**
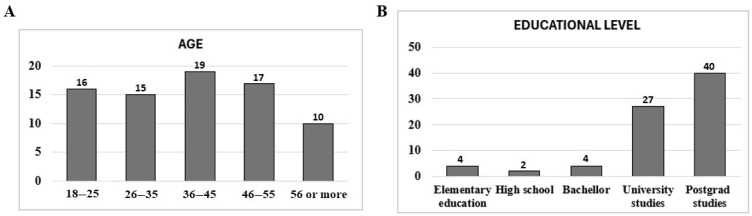
Statistical data society ranks. The bar graphs represent the percentage (number above bars) of participants in the survey. (**A**) Range of ages for the participants. (**B**) Educational level of the participants.

The citizens who participated in the optional survey (N = 77) ranged in ages from 19 to 78 years, with balanced participation among the different age ranges (Figure 4A). However, the educational level showed a clear deviation towards high educational levels (university and postgrad studies for 87% of the participants; Figure 4B).

**Figure 5:**
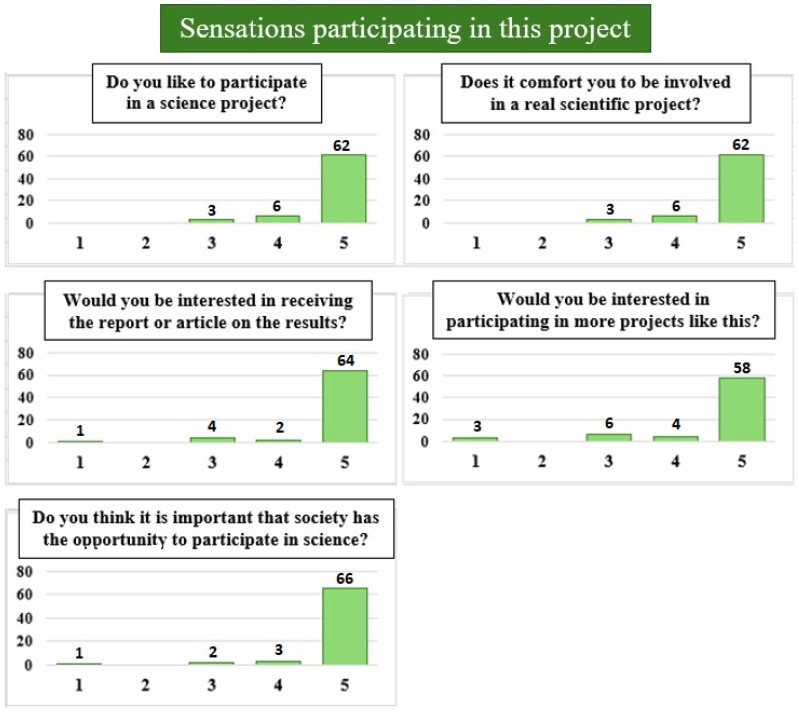
Responses about participants’ feelings regarding the project are presented on a scale from 1 to 5 (*x*-axis). A value of 1 indicates “I do not like it,” “uncomfortable,” “not interested,” or “not important,” while a value of 5 represents “I like it,” “very comfortable,” “very interested,” or “very important”. The bar graph (*y*-axis) represents the percentages of responses (exact % numbers are shown above them).

**Figure 6:**
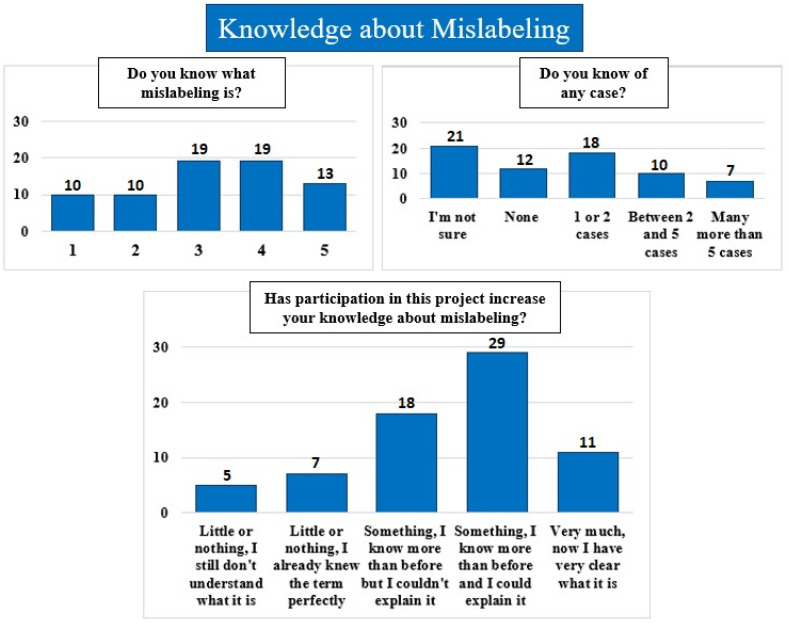
Responses regarding knowledge about mislabeling before and after participating in the project. The bar graph shows the percentage (numbers above bars) distribution of answers (*y*-axis). In the upper left graph (“Do you know what mislabeling is?”), a value of 1 indicates “I don’t know anything,” while a value of 5 indicates “I am an expert.”.

When we asked about their feelings about taking part in this project (Figure 5), all the answers were very positive, with more than 87% of the answers having the highest value in all questions. This suggests that having the opportunity to be involved in real scientific projects is very well received and valued by society. When we specifically asked about the mislabeling term (Figure 6), the responses were more varied, although just over half (53.5%) reported a medium or medium-high level of knowledge of the term. In addition, 51.5% claimed to know at least one case of mislabeling and 10.3% mentioned knowing more than five cases. Finally, when we asked if after participation in the project their knowledge about mislabeling had increased, 82.9% reported an improvement in their knowledge of the term, among which 41.4% stated that they are now able to explain it to other people and 15.7% felt sure that they understand the concept very clearly.

Finally, at the end of the survey, three questions related to the current relationship between science and society were included, with the intention of evaluating whether the contact and transfer of information between science and society is sufficient and efficient, from the point of view of the citizens (Supporting Materials S3: Figure S2). In this case, the answers showed a clear problem with the current level of contact and the transfer of information. A total of 97.1% answered that the contact is scarce and the transfer is limited. Furthermore, 73.2% expressed that they would like to have more contact and transfer and that they consider it essential and this should be in both directions, from the scientific community to society and vice versa. Lastly, we asked them how this contact and transfer of information between the scientific community and society could be improved (Supporting Materials S3: Figure S2), and the options most repeated were dissemination on social networks (24.4%), interviews or news on TV and radio (22.6%), and informative talks on new projects and results in public organizations and specific associations (19.7%), all together being 66.7% of the answers. Additionally, four participants proposed new options through the “others” option: to dedicate more money to research as the way to increase the capacity of dissemination (1), to make documentaries on research (1), and to organize not only talks but participatory workshops in associations, schools, institutes and for the general population (2).

## 4. Discussion

Given the magnitude and extent of the food fraud problem and its constant growth, the search for solutions to be able to carry out greater control in a more effective and rigorous manner is essential to maintain a trustworthy market where the health and rights of the consumer are protected [2]. The knowledge of the risk and vulnerability of food products that are marketed and consumed is the best way to effectively analyze and decide on the best fraud prevention and mitigation strategy [29]. Here we have evaluated the potential of Citizen Science and the involvement of society in the study and control of the risk of food fraud, encouraging volunteers to act as samplers collecting squid samples from Spanish bars and restaurants to figure out the current mislabeling rate in the *Loligo* spp. products offered.

### 4.1. Success and Quality of Sampling Based on Citizen Science

We found a bias regarding the level of education in the volunteers, 87% of them having a university level of education or higher. This could be related to the recruitment of participants mainly from our close circle. However, it is not such a large bias when one considers that, according to the report “Data and Figures of the Spanish University System 2022–2023” prepared by the Spanish Ministry of Universities, 40% of the Spanish population between 25–64 years of age has university studies or higher.

Thanks to this citizen collaboration (79 citizens and 7 experts), the performed sampling was highly successful, with more than 100 samples collected with all required information (city, food establishment, date, name on menu, sample type, cooking, price, etc.) in a few months (93.55% of the samples were successfully recovered). Counting the frequency with complete information being returned has already been used as an indicator of credibility in other important studies like the Oceana campaign for seafood mislabeling in 2011 [16]. Our response rate was much higher than in that study (where it was 33.4%) and other previous studies (e.g., 38% [30]; 13% [31]), likely motivated by the majority of volunteers being close to the researchers, which must be taken into account for future work. Another factor that may have encouraged the submission of samples with all essential information included is the use of digital platforms, such as a QR code linked to an online form in our case. Studies such as that by Finger et al. [18] have already highlighted that the use of online forms boosts citizen participation. It is easier for volunteers to participate if they only must enter data via their smartphone than if they must carry a pen and paper to collect the information.

Another parameter used in that study to evaluate the data quality was the failure to generate a DNA result from the collected samples. In our case, we successfully achieved genetic results for 107 of the collected samples (92.24%), and when we checked that percentage for experts and volunteers separately it was similar. Although other factors could contribute to failure in amplification, this success in genetic analyses suggests effort by the participants in following the instructions (freezing as soon as possible until getting back the sample) to maintain the correct preservation of the sample for its subsequent analysis. The good quality of data recovered here accords with other works where the authors concluded that the use of CS to collect samples in this kind of mislabeling studies provides high reliability and credibility data (e.g., [16,18,32]).

Another important factor in evaluating the utility of the volunteers as samplers is the coverage of the territory needed. In this case, we got samples from all autonomous communities except three of them (Figure 3), and the samples came from 48 different locations, which allowed us to obtain a broad view of the current species of squid sold in Spanish restaurants and the geographic reach of the mislabeling problem for *Loligo* genus (discussed below), obtaining again very similar results of mislabeling when we analyzed the samples for experts and volunteers separately, which adds reliability to the data. The consistency observed between the datasets collected by experts and those derived from CS is consistent with the findings of previous scientific studies [18,33], demonstrating that their use yields high-quality data. On the other hand, as it has been highlighted in other works (e.g., [12,34]), obtaining samples from so many different sites would have been impossible to obtain solely through the resources available to competent authorities or designated professional scientists without the collaboration of citizens, entailing an expenditure of money on travel that would be impossible to afford.

Although we are aware that, ideally, the number of samples would have been higher if this were an official campaign from a government, we consider that the success in number and coverage of the sampling has been resounding for an initial screening to assess the extent of mislabeling in restaurants for this group of squid. The number of samples collected in Madrid—the country’s capital and the center of its market—was very high, and the samples collected in other locations were from localities highly diverse in terms of both geographic location within the country and population size, ranging from large cities like Vigo (a city of approximately 300,000 inhabitants) to small towns like Villaricos (a village of approximately 670 inhabitants). The bias caused by collecting samples only from large cities has previously been noted in CS studies [18], but in our case, a significant number of samples (31.3%) were collected from small towns and villages with low populations (<10,000 inhabitants), thus reducing it.

On the other hand, the use of citizens themselves as samplers has also provided additional information, such as the preferences of consumers when they choose at the restaurants between the types of presentation (plate or sandwich) as well as the cooking method (fried and battered, grilled or in its ink), making it clear that the preference leans towards the plate of fried and battered calamari despite being more expensive than the alternative of eating them in sandwiches. Although the mislabeling found for a plate compared to that found for a sandwich was not significant, it does seem to be somewhat greater in the case of consuming fried and battered squid (92.6% versus 71.4%), showing that this form of presentation, together with its high consumption (based on the consumer’s preferences), is more vulnerable to fraud.

### 4.2. Limitations and Future Recommendations Regarding the Use of CS for Obtaining New Datasets

We think it is also important to be critical in order to establish improvements in the sampling procedure based on CS that make the data obtained even more reliable. Our study, while providing valuable information on the squid actually consumed in Spanish restaurants, as well as the mislabeling rate within the *Loligo* genus, the most prized, has also had some limitations. Firstly, the scope of the project could have been broader to obtain a larger number of samples from some autonomous communities, excluding the Community of Madrid. This would have required greater media coverage, but unfortunately, we were unable to allocate a portion of the budget to this task. Secondly, having more delivery points distributed throughout the country or a larger budget for organizing shipments would have also increased participation. In this regard, working with samples that can deteriorate during shipping over time significantly complicates logistics. Because of all that, for this type of research, coordination between administration and scientists seems to be the right thing to do [35].

In order to involve the population in official campaigns coordinated with authorities and scientists, we would include the following suggestions:

- A broad recruitment of volunteers, reaching out to the population at all educational levels, as well as a wide sampling scheme in different types of restaurants and city areas. To this end, dissemination in the media and social networks seems essential, in line with what was expressed by the volunteers in this study, as well as informative talks and collaboration with specialized organizations that will help the study’s information reach more types of people. As described by Lin and Kant [36], social media has transformed citizen participation by providing accessible, real-time platforms that remove physical and time barriers. Their open and informal nature fosters social inclusion and empowers groups often excluded by traditional methods. Consequently, these tools enhance transparency and efficiency in the interaction between governments and citizens, leading to more collaborative and representative solutions.
- To encourage participation, only the completion of the sample information should be mandatory, while opinion and satisfaction surveys should always be optional and as short as possible so as not to take too much time for volunteers. Shorter questionnaires have been associated with higher response rates [37].
- As we have tested in this study, it is important to always have two sampler groups, one formed only by experts and another one only for volunteers, in order to check possible bias made by volunteers. This method of assessing data quality is widely accepted and has been implemented in numerous CS studies (e.g., [16,18]).
- Ask for photos of the menu and plate consumed, together with the number of the sample, to solve any questions about the food or label after the sampling and as extra verification points.

### 4.3. Mislabeling Found in Loligo spp. Squids in Spanish Restaurants

The squids are one of the most commercially important groups of cephalopods, occupying approximately 33.7% of their annual production [38]. Among them, “calamares”, which in Spain officially means high-quality squids belonging to *Loligo* spp., is the most appreciated. In this study, the mislabeling for this group was huge, with 91.59% of samples mislabeled (only nine out of 107 samples belonging to *Loligo* spp.). This rate of mislabeling is higher than the rate detected in other studies performed in Spain and other European countries for cephalopods or squids (where there were not only alleged *Loligo* samples), which ranged between 11.1% to 67.2% (e.g., [12,21,22,23,24,39,40,41]). However, it is consistent with the fact that in many of these studies, *Loligo* spp. was highlighted as one of the most affected by mislabeling and substitution, but in none of these works was this genus of squid evaluated alone, thus diluting the possible mislabeling detected for it. In fact, in the analysis carried out by Luque and Donlan [19] on the estimates of seafood mislabeling, they conclude that focusing studies on specific genera or species and particular products would offer more efficient and realistic estimations and our study is proof of that. Furthermore, the samples analyzed here were collected from restaurants, and several authors have already showed that food services, including restaurants, are more susceptible to fraudulent practices because they are at the end of the supply chain and it is not compulsory to detail the scientific names on the menus, making this sector more vulnerable to substitution [7,12]. Therefore, there is an increase in the mislabeling rate because that is also logical.

Mislabeling was lower in the coastal regions, agreeing with there being more accessibility to the local products than in inland provinces where it is necessary to import any species, these being more susceptible to exchanges on the way. In fact, the *Loligo* species detected are regularly fished on the Spanish coasts, both Atlantic and Mediterranean. When we focus on the species found as substitutes, only four species were detected, all of them cheaper and/or less appreciated than *Loligo* species ([12]; https://www.fao.org/in-action/globefish/prices/en/; last accessed on 22 October 2024), and none of them are native to the eastern Atlantic Ocean or the Mediterranean Sea (WoRMS). Among them, two, *I. argentinus* and *D. gigas*, are used clearly as the main substitutes in Spain (85.7% of substitutions). This is consistent with one of the most recent studies on processed squid products [24], where these two species were the most frequently declared on the labels of these products (68.9%), this being proportional to their reported global captures. Different species of *Illex* have been commonly found as substitutes of *Loligo* in other studies [12,22,39,40,41], being more appreciated and like *Loligo* spp. than *D. gigas*, and therefore easier to sneak in as a substitute. Consistent with this, this species was a little bit more present in the coastal regions, where the consumers tend to have more experience to recognize the species that they are consuming. In the case of *D. gigas*, this species has also been detected as a substitute for other cephalopods like octopus [12,22,40] although is very different from the *Loligo* species, with a bigger size and a tougher flesh [42], so it is logical that it appears more in inland regions, where seafood consumption is lower and the sellers offer their product to generally less experienced consumers. The other two species found as substitutes, *D. gahi* and *U. duvaucelii*, were only detected in three and two provinces respectively (Pontevedra, Asturias, and Almería; Figure 3), all of them provinces where the seafood consumption is really high. These two species belonged to *Loligo* genus some time ago, as *Loligo gahi* and *Loligo duvaucelii*, but these scientific names have been unaccepted for quite a few years now according to the WoRMS and Sealifebase databases. Hence, the use of these species as *Loligo* spp. is totally incorrect and should be considered mislabeling. According to that, in the Spanish list of the official common and scientific names for seafood, published by the Ministry of Agriculture, Fisheries and Food, *D. gahi* appears with the current correct name and should be identified as “calamar patagónico” or “calamar del Perú” on the menu. However, in the case of *U. duvaucelii*, we have detected an error since the Spanish government has not updated the correct scientific name and *Loligo duvaucelii* continues to appear, called “calamar de la India” or “puntilla de la India”. This can lead to errors in the purchase and sale of this species, and sellers may mistakenly consider it a *Loligo*. Therefore, this list must be updated urgently to ensure that all scientific names appearing on it are currently accepted for each of the species marketed, in order to prevent this type of confusion. In any case, if we considered the three samples of this species as correct, following the outdated Spanish nomenclature, the mislabeling would still be enormous (88.79% in general; 90.91% for experts and 88.24% for volunteers).

In addition to all this, the species sold that belonged to *Loligo* genus (the one officially associated with this common commercial name and therefore correctly labeled on the menu) showed higher prices on the menus, indicating that sellers are aware that they are selling a species of greater value or more appreciated and are therefore increasing their sale price. The main consequence for the consumer in this case is being deceived, receiving a product that, while indeed a squid, is not what they expected, being a lower-value product. Furthermore, this exchange can also lead to the mistaken perception of consuming a locally sourced species (understood as one caught in waters around Spain), instead of a distant product that has most likely been flash-frozen and will not be fresh. Therefore, its nutritional properties will also be somewhat different. On the other hand, these substitute species, fished in distant ocean areas, often offer less information about their fishing methods, and in many cases, their stocks are suspected of being stretched to their limits or overexploited [24]. If consumers do not know the current species that they are consuming, they are indirectly being prevented from making consumption choices based on sustainability and the protection of natural resources. The alleged intentionality in fraud, together with the high frequency with which it occurs, highlights the need to incorporate the scientific name also in the labeling of menus in food services, where is not currently mandatory. The use of commercial name instead of scientific name has been proposed as one of the major sources of mislabeling [43]. Particularly in squid, as shown in the article by Sotelo et al. [24], it is a major problem that favors ambiguity, confusion and the variety of species under the same name. This action will prevent the possibility of hiding the use of a species of lesser value behind a generic common name allowing intentional confusion of the consumer and preventing them from making an informed decision.

### 4.4. Citizen Science as Science–Society “Glue”

Citizen Science is a growing field [16] which has enormous potential to improve the relationship between science and society. Several studies (e.g., [14,18,34,44]) have already advertised that encouraging and allowing citizen participation in scientific projects leads to greater learning about the subject matter, as well as increased interest and confidence in science and its results. In this work, most volunteers (82.9%) claimed to have greater knowledge about mislabeling after participating in the project. Similarly, the vast majority of participants expressed their interest in knowing the results obtained (90.1%), the satisfaction they felt when participating in a real scientific project (87.3%), as well as their interest in participating in future projects (81.7%), highlighting the enormous importance of society having the opportunity to participate and be directly involved in science. According to the literature [18,35,36], CS significantly enhances scientific learning and interest in research by actively involving people in data collection and analysis processes and our results clearly support that claim.

The volunteers consulted in this work also demanded more contact and transference of information between science and society, showing the clear need for this contact to be in both directions, from scientific community to society and vice versa. In this sense, having the privilege of counting on the opinion and direct participation of volunteers allows for a more realistic reflection of the problems faced by consumers in their daily lives, encouraging the creation of measures that really solve or improve real problems and motivating greater citizen participation in decisions on important issues [34]. While this increase in communication and transfer between citizens and research is fundamental in any field, on the path towards food safety and food traceability it is essential, since authorities will hardly be able to achieve good market control without the involvement of those who are part of it and/or consume its products. As other authors have pointed out (e.g., [45]), it is important for the future of food control not only to work with society, but also to train it so that it can actively help in the detection and mitigation of food fraud, as well as in decision-making and the development of regulations, and then exercising its great power as the consumers through choices made when purchasing one product or another. Along this line, putting the importance of food control under the light of a real scientific project seems practical and logical via a good understanding by society of the magnitude of the food fraud problem and the potential solutions, as well as increasing the transference among researchers, policy makers and citizens.

Finally, the information about the current risk/vulnerability status of a specific product is also useful, not only for the competent authorities, but also for the food industry, which does not always have the resources and/or information to detect fraud or establish the correct mitigation measures [29]. In this sense, our study is a clear example of the huge potential of using CS in traceability studies, thanks to which we have described the mislabeling rate, its magnitude, and have detected the main substitutes of *Loligo* squid in Spanish restaurants. It is interesting to imagine what we can achieve in future traceability studies using CS, with more resources and a coordinated effort of science, government and society.

## 5. Conclusions

Citizen Science can be used as a great tool for traceability studies, being relevant mainly for two reasons: (i) facilitating big samplings covering wide areas (in this work almost a complete country such as Spain), and (ii) improving the general public awareness in relation to food control, the traceability of particular food products (*Loligo* squids here), and its importance. Specifically, here, thanks to citizen participation, we have estimated the mislabeling rate in *Loligo* spp. in Spain, which has been found to be extremely common (91.59% of samples analyzed) and higher than in other European countries. Consequently, its control must be reinforced by national authorities. The information obtained in a collaborative way between researchers and citizens will be useful for driving such control/policy, recommending a greater need for control in inland areas than in coastal localities, and in the north of Spain versus the south, focusing on a greater number of evaluations of the cheaper products where the mislabeling seems to be more frequent. Finally, the citizen involvement in this study on food mislabeling has also suggested an improvement in volunteers’ knowledge about this problem after their participation, contributing to a more informed society that knows how to make better use of its power as consumers.

## Figures and Tables

**Table 1:** Summary of sample prices: mean, median, range, and standard deviation (Std. Dev.). In cases where a statistical test (*t*-test) could be applied to compare prices across different categories, the result is presented in the last column. N: number of samples.

		Price (€)					*t*-Test
		Mean	Median	Range	Std. Dev.	N	*p*-Value
**Type of sample**	Plate	11.1	11.00	2–21	3.94	73	<0.0001
	Sandwich	5.84	5.25	3.5–11.5	1.99	24	
**Food labeling**	Correct	13.71	15.50	2–19	5.45	9	<0.005
	Incorrect	9.40	9.00	2–21	3.90	88	
**Food on plate**	Not mislabeled	14.43	15.50	2–19	5.35	8	<0.05
	Mislabeled	10.69	10.50	2–21	3.58	65	
**Food on sandwich**	Not mislabeled	8.00	8.00	---	---	1	
	Mislabeled	5.74	5.00	3.5–11.5	1.98	23	

## Data Availability

The original contributions presented in this study are included in the article/supplementary material. Further inquiries can be directed to the corresponding authors.

## Supplementary Materials

The following supporting information can be downloaded at: https://www.mdpi.com/article/10.3390/foods15101690/s1.
