# Supplementary material for: Can Citizen Science Be a Key Factor in the Fight Against Mislabeling? Discovering What Squid Is on the Plate
Source: Foods. 2026 May 12;15(10):1690. doi: 10.3390/foods15101690 (PMC13205509; doi:10.3390/foods15101690)
Supplement: Supplementary file 1 [file foods-15-01690-s001.zip › foods-4288894-supplementary.pdf]

Supporting Materials S1

(i) Statistical data society ranks:

- p1. AGE
- p2. EDUCATIONAL LEVEL

(Elementary education / high school / Bachelor / University studies / Postgrads studies)

(ii) Sensations participating in this project:

- p3. DO YOU LIKE TO PARTICIPATE IN A SCIENCE PROJECT?

(I do not like it) 1 2 3 4 5 (I like it too much)

- p4. DOES IT COMFORT YOU TO BE INVOLVED IN A REAL SCIENTIFIC PROJECT?

(I don't find it comforting at all) 1 2 3 4 5 (I find it very comforting)

- p5. WOULD YOU BE INTERESTED IN RECEIVING THE REPORT OR ARTICLE ON THE RESULTS?

(I wouldn't be interested in anything) 1 2 3 4 5 (I would be very interested)

- p6. WOULD YOU BE INTERESTED IN PARTICIPATING IN MORE PROJECTS LIKE THIS?

(Not interested) 1 2 3 4 5 (Very interested)

- p7. DO YOU THINK IT IS IMPORTANT THAT SOCIETY HAS THE OPPORTUNITY TO PARTICIPATE IN SCIENCE?

(Nothing important) 1 2 3 4 5 (very important)

(iii) Knowledge about Mislabeling

- p8. DO YOU KNOW WHAT MISLABELING IS?

(I don't know anything) 1 2 3 4 5 (I am an expert)

- p9. DO YOU KNOW OF ANY CASE?

(I'm not sure / None / 1 or 2 cases / Between 2 and 5 cases / Many more than 5 cases)

- p10. HAS PARTICIPATION IN THIS PROJECT INCREASE YOUR KNOWLEDGE ABOUT MISLABELING?

(Little or nothing, I still don't understand what it is / Little or nothing, I already knew the term perfectly / Something, I know more than before but I couldn't explain it / Something, I know more than before and I could explain it / Very much, now I have very clear what it is)

(iv) Assessment of the current relationship between science and society and possible actions

- p11. DO YOU THINK THERE IS ENOUGH CONTACT BETWEEN SCIENCE AND SOCIETY?

(There is hardly any contact and no transfer of information / I think there is very limited contact with hardly any transfer of information / I think there is basic contact with little transfer of information / I think there is continuous contact with transfer of information)

- p12. WOULD YOU LIKE THERE TO BE MORE TRANSFER OF INFORMATION BETWEEN THE SCIENTIFIC COMMUNITY AND SOCIETY?

(No, I believe that a greater transfer of information between the scientific community and society is not necessary / Yes, especially from society to the scientific community / Yes, especially from the scientific community to society / Yes, and I see it as essential in both senses, from the scientific community to society and vice versa)

- p13. WHICH OF THE FOLLOWING ACTIONS DO YOU THINK WOULD HELP IMPROVE THE RELATIONSHIP BETWEEN SCIENCE AND SOCIETY?

- Dissemination on social networks
- Interviews or news on TV and radio
- Notes and interviews in the press
- Public round tables
- Informative talks on new projects and results in public organizations and specific associations (universities, schools, Ministries, associations, etc.)
- Informative talks on new projects and results at fairs, celebrations, social leisure events, etc.
- Others:

Supporting Materials S2

Information about sampling numbers and localities.

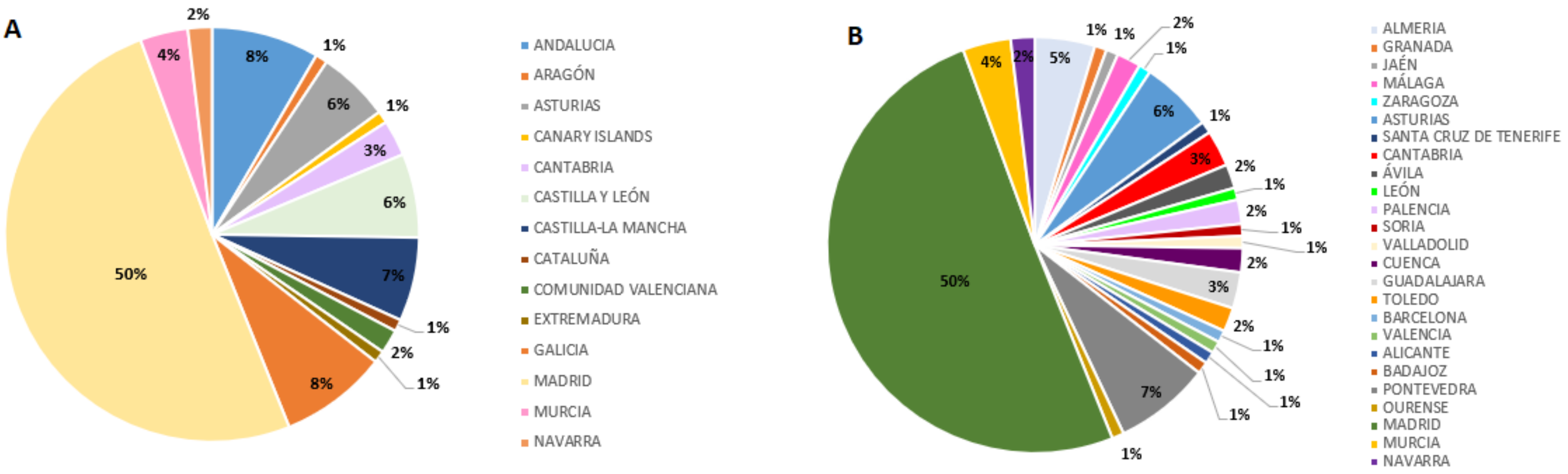

Fig. S1: Distribution of the 107 complete samples among Autonomous Communities (A) and Provinces (B) of Spain.

Table S1: locality, province, and autonomous community where the 107 samples considered "complete" were collected. In locations in which any sample result in *Loligo* spp. after genetic analyses are in bold and the number of *Loligo* spp. is indicated in parenthesis.

AUTONOMOUS COMMUNITY	N	PROVINCE	N	LOCATION	N
ANDALUCIA	9	ALMERIA	5	**San José**	**1(1)**
				Villaricos	1
				El Pozo de los Frailes	1
				Pozo del Esparto	2
		GRANADA	1	Castril	1
		JAÉN	1	Cazorla	1
		MÁLAGA	2	**Málaga**	**1(1)**
				**Marbella**	**1(1)**
ARAGÓN	1	ZARAGOZA	1	Zaragoza	1
ASTURIAS	6	ASTURIAS	6	Avilés	1
				Cudillero	1
				**Luarca**	**1(1)**
				**Piedras blancas**	**2(1)**
				Playa de Cueva	1
CANARY ISLANDS	1	SANTA CRUZ DE TENERIFE	1	Alcalá	1
CANTABRIA	3	CANTABRIA	3	Torrelavega	1
				Santander	2
CASTILLA Y LEÓN	7	ÁVILA	2	**Hoyo de Pinares**	**2(1)**
		LEÓN	1	León	1
		PALENCIA	2	Palencia	2
		SORIA	1	Covaleda	1
		VALLADOLID	1	Ataquines	1
CASTILLA-LA MANCHA	7	CUENCA	2	**Cuenca**	**2(1)**
		GUADALAJARA	3	Guadalajara	1
				Azuqueca de Henares	2
		TOLEDO	2	La Nava de Ricomalillo	1

				Toledo	1
CATALUÑA	1	BARCELONA	1	Barcelona	1
COMUNIDAD VALENCIANA	2	VALENCIA	1	Gandía	1
		ALICANTE	1	**Benidorm**	**1(1)**
EXTREMADURA	1	BADAJOZ	1	Zafra	1
GALICIA	9	PONTEVEDRA	8	Vigo	7
				Tomiño	1
		OURENSE	1	Padornelo	1
MADRID	54	MADRID	54	Alcorcon	1
				El Pardo	1
				Fuenlabrada	4
				Getafe	3
				Hoyo de Manzanares	1
				Leganés	1
				**Madrid**	**34(1)**
				Móstoles	1
				Rivas Vaciamadrid	6
				Torrejón de Ardóz	1
				Valdemoro	1
MURCIA	4	MURCIA	4	Cartagena	3
				Los Nietos	1
NAVARRA	2	NAVARRA	2	Pamplona	2

Table S2. BLAST results after genetic analyses. SOCN = Spanish Official Commercial name disclosed in the list of 2023. The *Loligo* spp. samples are marked in bold.

ID	First species match	Common name	SOCN	AN NCBI	Identity (%)	Cov. (%)		E-value	Second species match	Common name	Identity (%)	Cov. (%)	E-value
C1	*Dosidicus gigas*	Humboldt squid	"Potón del Pacífico"	KY446782.1	100	628/628	100	0.0	*Sthenoteuthis oualaniensis*	Purpleback flying squid	88.16	89	0.0
C2	*Dosidicus gigas*	Humboldt squid	"Potón del Pacífico"	KY446782.1	99.84	628/629	100	0.0	*Sthenoteuthis oualaniensis*	Purpleback flying squid	87.74	99	0.0
C3	*Illex argentinus*	Argentine shortfin squid	"Pota argentina"	NC_026908.1	100	617/617	100	0.0	*Illex coindetii*	Southern shortfin squid	97.06	99	0.0
C7	*Dosidicus gigas*	Humboldt squid	"Potón del Pacífico"	KY446782.1	100	599/599	99	0.0	*Sthenoteuthis oualaniensis*	Purpleback flying squid	88.10	99	0.0
C8	*Dosidicus gigas*	Humboldt squid	"Potón del Pacífico"	KY446782.1	100	588/588	99	0.0	*Sthenoteuthis oualaniensis*	Purpleback flying squid	88.25	99	0.0
C13	*Doryteuthis gahi*	Patagonian longfin squid	"Calamar patagónico o calamar del Perú"	KY771101.1	99.67	598/600	99	0.0	*Doryteuthis opalescens*	opalescent inshore squid	85.67	100	1,00E-180
C14	*Dosidicus gigas*	Humboldt squid	"Potón del Pacífico"	KY446782.1	100	627/627	100	0.0	*Sthenoteuthis oualaniensis*	Purpleback flying squid	87.74	100	0.0
C16	*Doryteuthis gahi*	Patagonian longfin squid	"Calamar patagónico o calamar del Perú"	MH194501.1	99.49	589/592	99	0.0	*Doryteuthis pelaei*	Longfin inshore squid	87.16	97	0.0
**C18**	***Loligo vulgaris***	**European squid or common squid**	**"Calamar o Calamar europeo"**	**MN977133.1**	**99.36**	**467/470**	**100**	**0.0**	***Loligo reynaudi***	**Cape Hope squid**	**95.32**	**100**	**0.0**
C22	*Dosidicus gigas*	Humboldt squid	"Potón del Pacífico"	KY446782.1	100	623/623	100	0.0	*Sthenoteuthis oualaniensis*	Purpleback flying squid	88.03	99	0.0

C25	*Dosidicus gigas*	Humboldt squid	"Potón del Pacífico"	KY446782.1	100	625/625	99.84	0.0	*Sthenoteuthis oualaniensis*	Purpleback flying squid	87.84	99	0.0
C26	*Illex argentinus*	Argentine shortfin squid	"Pota argentina"	NC_026908.1	99.84	614/615	100	0.0	*Illex coindetii*	Southern shortfin squid	96.89	99	0.0
C40	*Illex argentinus*	Argentine shortfin squid	"Pota argentina"	NC_026908.1	100	616/616	100	0.0	*Illex coindetii*	Southern shortfin squid	97.05	99	0.0
C42	*Illex argentinus*	Argentine shortfin squid	"Pota argentina"	DQ373957.1	100	611/611	98	0.0	*Nototodarus sloanii*	New Zealand arrow squid or Wellington flying squid	99.82	90	0.0
**C47**	***Loligo vulgaris***	**European squid or common squid**	**"Calamar o Calamar europeo"**	**KM517927.1**	**99.67**	**608/610**	**97**	**0.0**	***Loligo reynaudi***	**Cape Hope squid**	**94.64**	**92**	**0.0**
C61	*Illex argentinus*	Argentine shortfin squid	"Pota argentina"	DQ373957.1	100	602/602	97	0.0	*Nototodarus sloanii*	New Zealand arrow squid or Wellington flying squid	99.82	89	0.0
C72	*Dosidicus gigas*	Humboldt squid	"Potón del Pacífico"	KY446782.1	99.80	501/502	98	0.0	*Sthenoteuthis oualaniensis*	Purpleback flying squid	88.67	99	2,00E-171
**C74**	***Loligo vulgaris***	**European squid or common squid**	**"Calamar o Calamar europeo"**	**KM517927.1**	**100**	**555/555**	**95**	**0.0**	***Loligo reynaudi***	**Cape Hope squid**	**95.32**	**95**	**0.0**
C75	*Dosidicus gigas*	Humboldt squid	"Potón del Pacífico"	KY446782.1	100	589/589	100	0.0	*Sthenoteuthis oualaniensis*	Purpleback flying squid	84,14	100	0.0
C81	*Doryteuthis gahi*	Patagonian longfin squid	"Calamar patagónico	MH194501.1	99.67	611/613	99	0.0	*Doryteuthis opalescens*	opalescent inshore squid	85.69	100	0.0

			o calamar del Perú"										
C83	*Dosidicus gigas*	Humboldt squid	"Poton del Pacífico"	KY446782.1	99.68	624/626	100	0.0	*Sthenoteuthis oualaniensis*	Purpleback flying squid	87.84	99	0.0
C84	*Illex argentinus*	Argentine shortfin squid	"Pota argentina"	NC_026908.1	100	615/615	100	0.0	*Illex coindetii*	Southern shortfin squid	96.89	99	0.0
C87	*Illex argentinus*	Argentine shortfin squid	"Pota argentina"	NC_026908.1	100	617/617	100	0.0	*Illex coindetii*	Southern shortfin squid	96.90	99	0.0
C88	*Illex argentinus*	Argentine shortfin squid	"Pota argentina"	NC_026908.1	99.51	613/616	99	0.0	*Illex coindetii*	Southern shortfin squid	97.05	99	0.0
C89	*Illex argentinus*	Argentine shortfin squid	"Pota argentina"	NC_026908.1	100	614/614	100	0.0	*Illex coindetii*	Southern shortfin squid	96.88	99	0.0
C93	*Illex argentinus*	Argentine shortfin squid	"Pota argentina"	DQ373957.1	99.84	609/610	97	0.0	*Nototodarus sloanii*	New Zealand arrow squid or Wellington flying squid	100	90	0.0
C100	*Doryteuthis gahi*	Patagonian longfin squid	"Calamar patagónico o calamar del Perú"	MH194501.1	99.67	611/613	99	0.0	*Doryteuthis opalescens*	opalescent inshore squid	85.69	100	0.0
C102	*Doryteuthis gahi*	Patagonian longfin squid	"Calamar patagónico o calamar del Perú"	MH194501.1	99.67	611/613	99	0.0	*Doryteuthis opalescens*	opalescent inshore squid	85.67	100	1,00E-180
C103	*Doryteuthis gahi*	Patagonian longfin squid	"Calamar patagónico	MH194501.1	99.67	611/613	99	0.0	*Doryteuthis opalescens*	opalescent inshore squid	85.69	100	0.0

			o calamar del Perú"										
C104	*Illex argentinus*	Argentine shortfin squid	"Pota argentina"	NC_026908.1	99.57	465/467	100	0.0	*Illex coindetii*	Southern shortfin squid	96.54	98	0.0
C106	*Doryteuthis gahi*	Patagonian longfin squid	"Calamar patagónico o calamar del Perú"	MH194501.1	99.67	611/613	99	0.0	*Doryteuthis opalescens*	opalescent inshore squid	85.74	100	0.0
C107	*Dosidicus gigas*	Humboldt squid	"Potón del Pacífico"	KY446782.1	100	626/626	100	0.0	*Sthenoteuthis oualaniensis*	Purpleback flying squid	87.86	99	0.0
C109	*Uroteuthis duvauceli*	*Indian Ocean squid*		KP976398.1	100	621/621	99	0.0	*Uroteuthis chinensis*	Hanchi or Taiwanese squid or mitre squid	92.41	99	0.0
C115	*Dosidicus gigas*	Humboldt squid	"Potón del Pacífico"	KY446782.1	99.84	624/625	100	0.0	*Sthenoteuthis oualaniensis*	Purpleback flying squid	87.68	99	0.0
C116	*Doryteuthis gahi*	Patagonian longfin squid	"Calamar patagónico o calamar del Perú"	MH194501.1	99.67	606/608	99	0.0	*Doryteuthis opalescens*	opalescent inshore squid	85.74	99	4,00E-180
**C122**	***Loligo forbesii***	**veined squid and long-finned squid**	**"calamar veteado"**	**KM517907.1**	**100**	**609/609**	**97**	**0.0**	***Loligo vulgaris***	**European squid or common squid**	**91.30**	**97**	**0.0**
C123	*Illex argentinus*	Argentine shortfin squid	"Pota argentina"	NC_026908.1	100	616/616	100	0.0	*Illex coindetii*	Southern shortfin squid	96.89	99	0.0
C127	*Dosidicus gigas*	Humboldt squid	"Potón del Pacífico"	KY446782.1	100	626/626	100	0.0	*Sthenoteuthis oualaniensis*	Purpleback flying squid	87.84	99	0.0
C128	*Dosidicus gigas*	Humboldt squid	"Potón del Pacífico"	MH194426.1	99.57	464/466	100	0.0	*Sthenoteuthis oualaniensis*	Purpleback flying squid	88.60	99	6,00E-157

C129	*Dosidicus gigas*	Humboldt squid	"Potón del Pacífico"	KY446782.1	100	628/628	100	0.0	*Sthenoteuthis oualaniensis*	Purpleback flying squid	87.76	100	0.0
**C133**	***Loligo vulgaris***	**European squid or common squid**	**"Calamar o Calamar europeo"**	**KM517927.1**	**100**	**607/607**	**95**	**0.0**	***Loligo reynaudi***	**Cape Hope squid**	**94.96**	**90**	**0.0**
C137	*Dosidicus gigas*	Humboldt squid	"Potón del Pacífico"	KY446782.1	100	625/625	100	0.0	*Sthenoteuthis oualaniensis*	Purpleback flying squid	88.03	98	0.0
C138	*Dosidicus gigas*	Humboldt squid	"Potón del Pacífico"	KY446782.1	100	628/628	100	0.0	*Sthenoteuthis oualaniensis*	Purpleback flying squid	87.88	99	0.0
C143	*Illex argentinus*	Argentine shortfin squid	"Pota argentina"	DQ373957.1	99.83	601/602	95	0.0	*Nototodarus sloanii*	New Zealand arrow squid or Wellington flying squid	99.64	88	0.0
C144	*Dosidicus gigas*	Humboldt squid	"Potón del Pacífico"	KY446782.1	99.84	626/627	100	0.0	*Sthenoteuthis oualaniensis*	Purpleback flying squid	88.00	99	0.0
C145	*Illex argentinus*	Argentine shortfin squid	"Pota argentina"	NC_026908.1	100	615/615	100	0.0	*Illex coindetii*	Southern shortfin squid	97.05	99	0.0
C147	*Illex argentinus*	Argentine shortfin squid	"Pota argentina"	NC_026908.1	100	614/614	100	0.0	*Illex coindetii*	Southern shortfin squid	97.04	99	0.0
C150	*Dosidicus gigas*	Humboldt squid	"Potón del Pacífico"	KY446782.1	99.84	625/626	100	0.0	*Sthenoteuthis oualaniensis*	Purpleback flying squid	88.00	99	0.0
C155	*Dosidicus gigas*	Humboldt squid	"Potón del Pacífico"	KY446782.1	100	624/624	100	0.0	*Sthenoteuthis oualaniensis*	Purpleback flying squid	88.03	98	0.0
**C159**	***Loligo forbesii***	**veined squid and long-finned squid**	**"calamar veteado"**	**KM517907.1**	**100**	**602/602**	**97**	**0.0**	***Loligo vulgaris***	**European squid or common squid**	**91.20**	**97**	**0.0**

C160	*Dosidicus gigas*	Humboldt squid	"Potón del Pacífico"	KY446795.1	100	623/623	100	0.0	*Sthenoteuthis oualaniensis*	Purpleback flying squid	88.03	99	0.0
**C162**	***Loligo forbesii***	**veined squid and long-finned squid**	**"calamar veteado"**	**MT919766.1**	**99.84**	**610/611**	**97**	**0.0**	***Loligo vulgaris***	**European squid or common squid**	**91.33**	**97**	**0.0**
C166	*Dosidicus gigas*	Humboldt squid	"Potón del Pacífico"	KY446782.1	99.84	624/625	100	0.0	*Sthenoteuthis oualaniensis*	Purpleback flying squid	87.68	99	0.0
C169	*Doryteuthis gahi*	Patagonian longfin squid	"Calamar patagónico o calamar del Perú"	MH194501.1	99.67	608/610	99	0.0	*Doryteuthis opalescens*	opalescent inshore squid	85.76	99	1,00E-180
C170	*Illex argentinus*	Argentine shortfin squid	"Pota argentina"	DQ373957.1	100	573/573	95	0.0	*Nototodarus sloanii*	New Zealand arrow squid or Wellington flying squid	99.81	87	0.0
C173	*Illex argentinus*	Argentine shortfin squid	"Pota argentina"	NC_026908.1	99,84	614/615	100	0.0	*Illex coindetii*	Southern shortfin squid	96.89	99	0.0
C174	*Illex argentinus*	Argentine shortfin squid	"Pota argentina"	NC_026908.1	99.35	611/615	100	0.0	*Illex coindetii*	Southern shortfin squid	96.89	99	0.0
C176	*Dosidicus gigas*	Humboldt squid	"Potón del Pacífico"	KY446782.1	100	627/627	100	0.0	*Sthenoteuthis oualaniensis*	Purpleback flying squid	87.74	100	0.0
C182	*Illex argentinus*	Argentine shortfin squid	"Pota argentina"	NC_026908.1	100	617/617	100	0.0	*Illex coindetii*	Southern shortfin squid	97.06	99	0.0
C187	*Illex argentinus*	Argentine shortfin squid	"Pota argentina"	NC_026908.1	99.84	614/615	100	0.0	*Illex coindetii*	Southern shortfin squid	96.89	99	0.0

C193	*Dosidicus gigas*	Humboldt squid	"Potón del Pacífico"	KY446782.1	100	491/491	100	0.0	*Sthenoteuthis oualaniensis*	Purpleback flying squid	88.80	100	2,00E-167
C196	*Illex argentinus*	Argentine shortfin squid	"Pota argentina"	NC_026908.1	100	616/616	100	0.0	*Illex coindetii*	Southern shortfin squid	97.05	99	0.0
C199	*Dosidicus gigas*	Humboldt squid	"Potón del Pacífico"	MH194426.1	100	616/616	100	0.0	*Sthenoteuthis oualaniensis*	Purpleback flying squid	87.84	100	0.0
C200	*Illex argentinus*	Argentine shortfin squid	"Pota argentina"	NC_026908.1	99.84	615/616	100	0.0	*Illex coindetii*	Southern shortfin squid	96.89	99	0.0
C241	*Illex argentinus*	Argentine shortfin squid	"Pota argentina"	NC_026908.1	100	617/617	100	0.0	*Illex coindetii*	Southern shortfin squid	96.90	99	0.0
C251	*Illex argentinus*	Argentine shortfin squid	"Pota argentina"	DQ373957.1	99.84	609/610	96	0.0	*Nototodarus sloanii*	New Zealand arrow squid or Wellington flying squid	99.65	89	0.0
C252	*Dosidicus gigas*	Humboldt squid	"Potón del Pacífico"	KY446782.1	100	625/625	100	0.0	*Sthenoteuthis oualaniensis*	Purpleback flying squid	87.84	99	0.0
C253	*Dosidicus gigas*	Humboldt squid	"Potón del Pacífico"	KY446782.1	100	626/626	100	0.0	*Sthenoteuthis oualaniensis*	Purpleback flying squid	87.86	99	0.0
C255	*Illex argentinus*	Argentine shortfin squid	"Pota argentina"	DQ373957.1	100	609/609	96	0.0	*Nototodarus sloanii*	New Zealand arrow squid or Wellington flying squid	99.82	89	0.0
**C262**	**Loligo vulgaris**	**European squid or common squid**	**"Calamar o Calamar europeo"**	**MN977133.1**	**99.65**	**575/577**	**99**	**0.0**	**Loligo reynaudi**	**Cape Hope squid**	**94.97**	**99**	**0.0**
C267	*Dosidicus gigas*	Humboldt squid	"Potón del Pacífico"	MH194426.1	99.84	614/615	100	0.0	*Sthenoteuthis oualaniensis*	Purpleback flying squid	87.66	100	0.0

C270	*Illex argentinus*	Argentine shortfin squid	"Pota argentina"	NC_026908.1	100	616/616	100	0.0	*Illex coindetii*	Southern shortfin squid	97.05	99	0.0
C280	*Illex argentinus*	Argentine shortfin squid	"Pota argentina"	NC_026908.1	99.84	616/617	100	0.0	*Illex coindetii*	Southern shortfin squid	96.90	99	0.0
C281	*Dosidicus gigas*	Humboldt squid	"Potón del Pacífico"	MH194426.1	100	615/615	100	0.0	*Sthenoteuthis oualaniensis*	Purpleback flying squid	87.82	100	0.0
C288	*Illex argentinus*	Argentine shortfin squid	"Pota argentina"	NC_026908.1	99.84	614/615	100	0.0	*Illex coindetii*	Southern shortfin squid	96.89	99	0.0
C300	Illex argentinus	Argentine shortfin squid	"Pota argentina"	NC_026908.1	99.84	614/615	100	0.0	Illex coindetii	Southern shortfin squid	96.89	99	0.0
C301	*Dosidicus gigas*	Humboldt squid	"Potón del Pacífico"	MH194426.1	100	615/615	100	0.0	*Sthenoteuthis oualaniensis*	Purpleback flying squid	87.82	100	0.0
C311	*Illex argentinus*	Argentine shortfin squid	"Pota argentina"	NC_026908.1	99.84	614/615	100	0.0	*Illex coindetii*	Southern shortfin squid	96.89	99	0.0
C312	*Illex argentinus*	Argentine shortfin squid	"Pota argentina"	DQ373957.1	100	606/606	97	0.0	*Nototodarus sloanii*	New Zealand arrow squid or Wellington flying squid	99.82	90	0.0
C315	Illex argentinus	Argentine shortfin squid	"Pota argentina"	NC_026908.1	99.35	612/616	100	0.0	Illex coindetii	Southern shortfin squid	97.05	99	0.0
C317	*Illex argentinus*	Argentine shortfin squid	"Pota argentina"	NC_026908.1	99.84	611/612	100	0.0	*Illex coindetii*	Southern shortfin squid	96.87	99	0.0

C318	*Illex argentinus*	Argentine shortfin squid	"Pota argentina"	NC_026908.1	99.84	614/615	100	0.0	*Illex coindetii*	Southern shortfin squid	96.89	99	0.0
C335	*Doryteuthis gahi*	Patagonian longfin squid	"Calamar patagónico o calamar del Perú"	MH194501.1	99.84	612/613	99	0.0	*Doryteuthis opalescens*	opalescent inshore squid	85.48	99	2,00E-178
C337	Illex argentinus	Argentine shortfin squid	"Pota argentina"	NC_026908.1	100	575/575	100	0.0	Illex coindetii	Southern shortfin squid	96.84	99	0.0
C338	Dosidicus gigas	Humboldt squid	"Potón del Pacífico"	KY446782.1	99.84	625/626	100	0.0	Sthenoteuthis oualaniensis	Purpleback flying squid	88.00	99	0.0
C342	Uroteuthis duvauceli	Indian Ocean squid		KC951888.1	99.84	620/621	99	0.0	Uroteuthis chinensis	Hanchi or Taiwanese squid or mitre squid	92.74	99	0.0
C346	*Dosidicus gigas*	Humboldt squid	"Potón del Pacífico"	KY446795.1	99.68	623/625	100	0.0	*Sthenoteuthis oualaniensis*	Purpleback flying squid	87.86	100	0.0
C359	*Illex argentinus*	Argentine shortfin squid	"Pota argentina"	NC_026908.1	100	615/615	100	0.0	*Illex coindetii*	Southern shortfin squid	97.05	99	0.0
C360	Illex argentinus	Argentine shortfin squid	"Pota argentina"	DQ373957.1	99.84	610/611	97	0.0	Nototodarus sloanii	New Zealand arrow squid or Wellington flying squid	99.65	90	0.0
C362	Illex argentinus	Argentine shortfin squid	"Pota argentina"	NC_026908.1	99.84	615/616	100	0.0	Illex coindetii	Southern shortfin squid	96.89	99	0.0
C371	*Doryteuthis gahi*	Patagonian longfin squid	"Calamar patagónico o calamar del Perú"	MH194501.1	99.84	612/613	99	0.0	*Doryteuthis opalescens*	opalescent inshore squid	85.50	99	5,00E-179

C375	*Illex argentinus*	Argentine shortfin squid	"Pota argentina"	NC_026908.1	99.84	615/616	100	0.0	Illex coindetii	Southern shortfin squid	96.86	99	0.0
C376	*Illex argentinus*	Argentine shortfin squid	"Pota argentina"	NC_026908.1	100	616/616	100	0.0	Illex coindetii	Southern shortfin squid	97.05	99	0.0
C379	*Dosidicus gigas*	Humboldt squid	"Potón del Pacífico"	KY446782.1	100	587/587	100	0.0	Sthenoteuthis oualaniensis	Purpleback flying squid	88.25	99	0.0
C386	*Illex argentinus*	Argentine shortfin squid	"Pota argentina"	NC_026908.1	100	564/564	100	0.0	Illex coindetii	Southern shortfin squid	96.94	98	0.0
C387	*Dosidicus gigas*	Humboldt squid	"Potón del Pacífico"	KY446782.1	100	624/624	100	0.0	*Sthenoteuthis oualaniensis*	Purpleback flying squid	88.03	98	0.0
C389	Dosidicus gigas	Humboldt squid	"Potón del Pacífico"	KY446782.1	100	626/626	100	0.0	Sthenoteuthis oualaniensis	Purpleback flying squid	87.86	99	0.0
C390	Uroteuthis duvauceli	Indian Ocean squid		KP976398.1	99.63	539/541	100	0.0	Uroteuthis chinensis	Hanchi or Taiwanese squid or mitre squid	93.15	99	0.0
C395	*Dosidicus gigas*	Humboldt squid	"Potón del Pacífico"	KY446787.1	99.84	623/624	100	0.0	*Sthenoteuthis oualaniensis*	Purpleback flying squid	87.84	100	0.0
C406	Illex argentinus	Argentine shortfin squid	"Pota argentina"	NC_026908.1	99.84	615/616	100	0.0	Illex coindetii	Southern shortfin squid	96.89	99	0.0
C410	Illex argentinus	Argentine shortfin squid	"Pota argentina"	NC_026908.1	99.83	583/584	100	0.0	Illex coindetii	Southern shortfin squid	96.72	99	0.0
C413	*Dosidicus gigas*	Humboldt squid	"Potón del Pacífico"	KY446782.1	100	628/628	100	0.0	*Sthenoteuthis oualaniensis*	Purpleback flying squid	87.76	100	0.0
C414	Dosidicus gigas	Humboldt squid	"Potón del Pacífico"	KY446782.1	99.83	586/587	100	0.0	Sthenoteuthis oualaniensis	Purpleback flying squid	88.42	99	0.0

**C422**	**Loligo vulgaris**	**European squid or common squid**	**"Calamar o Calamar europeo"**	**MN977133.1**	**100**	**610/610**	**97**	**0.0**	**Loligo reynaudi**	**Cape Hope squid**	**95.08**	**97**	**0.0**
C429	*Dosidicus gigas*	Humboldt squid	"Potón del Pacífico"	KY446782.1	99.84	621/622	100	0.0	*Sthenoteuthis oualaniensis*	Purpleback flying squid	88.03	99	0.0
C436	Illex argentinus	Argentine shortfin squid	"Pota argentina"	NC_026908.1	99.84	613/614	100	0.0	Illex coindetii	Southern shortfin squid	96.88	99	0.0
C437	Illex argentinus	Argentine shortfin squid	"Pota argentina"	NC_026908.1	99.35	611/615	100	0.0	Illex coindetii	Southern shortfin squid	97.05	99	0.0

Supporting Materials S3

Assessment of the current relationship between science and society and possible actions

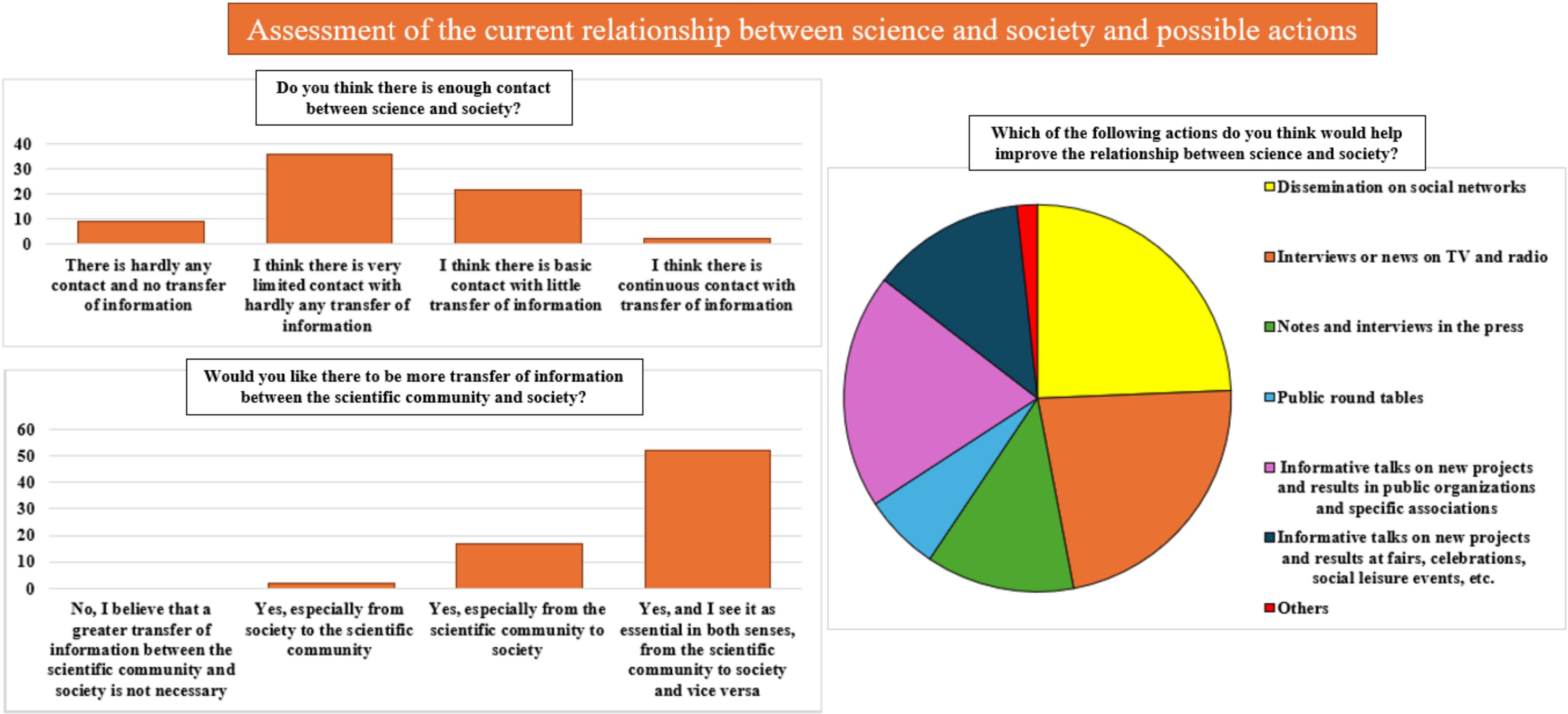

Fig. S2: Answers related to the current relationship between science and society. In the bar graphs, Y axis shows the number of answers.
